# Hospital and Institutional News

**Published:** 1919-08-16

**Authors:** 


### HOSPITAL AND INSTITUTIONAL NEWS.

### REGIONAL PENSIONS SYSTEM.

When recently meeting at Manchester the representatives of the War Pensions Committees of Lancashire, Cheshire, Westmorland and the Isle of Man, Sir Worthington Evans, Minister of Pensions, explained that there had been a second reading of the Bill, which would create a statutory right to a pension. According to his scheme tribunals were to be set up by Act of Parliament, so that any man, widow, or dependant would have a statutory right to go to an independent court, not controlled by the Ministry of Pensions, and their pension claim would be adjudicated by that court, whose decisions would be binding upon the Ministry of Pensions. The country would be divided into about thirteen districts, with Manchester as the Headquarters of the North-Western District, representing thus about 200,000 pensions, and in this way it was expected that the work would be done far better than by distant London conferences. The regional scheme, with regional control, appears most promising. Also the scheme provides that each region shall have its own medical service branch, and in the matter of medical assessments the old system, under which a board which had not seen the man was able to alter his award, will be abolished, each region having its own personal control. The Minister's speech contained, apart from the above, many suggestions of which we heartily approve, and his words forecast a number of urgently needed reforms.

### AFTER THE HOLIDAYS.

Since it is generally admitted that the best season in which to issue appeals is after the summer holidays, we hope that the many hospitals on the brink of an appeal will not lose the coming opportunity. This year, in particular, the chance should not be missed, for the winter is likely to draw everyone's purse-strings closer. High prices are expected. Labour troubles may revive, and the consequence is that Christmas appeals, which some people prefer, may prove disappointing. Voluntary hospital finance needs special forethought at the moment and the old reminder is doubly necessary.

### PREVALENCE OF FLEAS.

Dr. Hamer, the school medical officer for London, provides in his annual report some striking figures, showing the relationship between the scarlet fever returns and the prevalence of fleas. The latter was estimated by the number of children found to be bitten, and from the signs in common lodging-house beds. 1910 showed a slight increase in fleas and a slight increase in scarlet fever. In 1911 both figures declined, rising again in 1912. Then a marked rise in flea prevalence occurred in 1913-14-15, a noticeable increase in scarlet fever running exactly parallel with it. It has been pointed out that scarlet fever is not necessarily a dirt disease and has even a reputation as a condition of the well-to-do, but Dr. Hamer suggests that as far as young children, one to five years, are concerned the infection is far more frequent among the poor. In older children the prevalence is about equal irrespective of class. The conclusion is drawn that the facts as set out do not in any way conflict with acceptance of a flea hypothesis of spread of scarlet fever, provided it is borne in mind that there is undoubted evidence to show that in the poorer areas the disease tends to attack children at a younger age than in well-to-do areas, and thus renders the poorer children at the higher ages immune from attack.

### NEEDLES IN THE HEART.

An inquest was held in York on August 6 on a lady, a doctor of medicine, and latterly a patient in the Retreat, York. On November 17 last the deceased had told a visiting relative that she had attempted to kill herself and ought to be dead, since she had stuck a needle into her heart. The medical superintendent was informed and found two apparently simple pin pricks below the left breast. The patient had been suffering from melancholia, but grew very much better and her delusions left her. Nothing more was heard of the needles until quite recently, when, after a few days' illness, the lady died. A *post-mortem* examination showed one needle sticking through and a second one into the walls of the heart. Although signs of active inflammation were thus finally found, it is most surprising that no earlier evidence of the cardiac injury appeared. Had the position of the needles been realised at first, it is still doubtful whether they could have been extracted without the gravest immediate danger to the patient. The case strikes one as most unusual and remarkable, particularly in the length of its history.

### MEDICAL EXAMINATIONS FOR THE CLERGY.

Although medical examination is a *sine qua non* in many civil appointments, it is rarely if ever a necessary condition in the appointment of a clergyman to any office except that of Army and Naval chaplains. But advertising in the *Church Times* of August 1 for a chaplain for the Cane Hill Mental Hospital, the London County Council states that the "appointment [is] subject to medical examination."

### HERTFORDSHIRE SANITARY REPORT.

This twenty-first annual report comprises records of work done during 1918 in general sanitary measures, anti-tuberculosis, and school health; the two latter are given in separate brochures. It is significant that the birth-rate for that year has fallen below the death-rate. The M.O.H. notes (under Housing and Town Planning) that "from the point of view of public health in the county now, but more especially in the future, it is eminently desirable that any great demand for the provision of housing accommodation for London's population should be met by a comprehensive scheme of town planning." This recommendation should meet with approval in a county which contains the example of Letchworth. The death-rate in tuberculosis is found in many places to exceed the notification-rate, showing that some practitioners are neglecting to notify. An increase in the remuneration for this obligation is suggested. Malnutrition among children is found to be slightly less than in 1917, owing to some improvement in the food supply. A high percentage of unvaccinated children is again reported from all parts of the country, a matter causing concern in view of the number of troops returning from infected places abroad.

### WAR SAVINGS.

The popularity of War Savings Certificates was shown from the very commencement of the scheme in February 1916, and since then 280,701,054 separate Certificates have been issued. In the broken year of 1916 the sale of Certificates produced 42 millions; in 1917, 67 millions; and, in 1918, 108 millions. The organisation set up by the National War Savings Committee is very widespread, and full details of it are given in the third annual report of the Committee. Although we see on all sides evidence of unnecessary spending of money, it is evident that there are those who are wise enough to put by something until a time when they will get more value for their money. During 1918, in respect of deposits in the Post Office and Trustee Savings Banks, on purchases of War Loan through post offices, and on purchases of War Savings Certificates, the small investor placed £432,000,000 to his credit, as compared with £253,000,000 in 1917.

### KNOW ALL THE FACTS.

A correspondent in the *Daily Mail* for August 2 writes: "It is easier to work hard than to die hard." This, we think, is a fallacy; but it is one which is generally believed. No man likes to die, but in the enthusiasm of the war he started off, without the idea engrained on his sub-conscious mind of what it meant. When he got there and really realised it, he had no time to think, and when he found his back to the wall and knew he had "to die hard," we doubt whether it was very difficult. He only had to do it once. But the need to work hard comes again and again. The enthusiasm of the peace leads one to rest rather than energy; when the determination to work hard has been made it may carry us through a day, a week, a month, and then, as we become tired, it will ebb way. We shall have to go on for years. It will be very very hard, as the *Daily Mail* said in its heading to its correspondent's letter, "Let the people know all the facts," and let us try to realise how difficult it may be.

### DR. KING BROWN AND UNREGISTERED DENTISTS.

Bermondsey Borough Council, acting on the advice of Dr. King Brown, their medical officer of health, has decided to ask the Ministry of Health to promote legislation dealing with unqualified dentists. Dr. Brown pointed out the great evils associated with the unqualified practice of dentistry, though the supply of qualified dentists is at present insufficient. The main causes of the shortage are the unsatisfactory state of the law in regard to unqualified practice, and the duration and cost of training for a dental qualification. To meet the evils the legal prohibition of dental practice of unqualified persons is essential. He suggested that, under prescribed conditions, unregistered persons who have reputably practised dentistry for five years should be, in the event of prohibition being enforced, admitted to registration as dentists or dental assistants by a special *ad hoc* committee.

### AN INGENIOUS SLUM CELEBRATION.

Since no public entertainment in celebration of Peace was provided for the children of the district, the inhabitants of one of the slum parts of Walthamstow resolved to take the matter into their own hands. The men in each street formed themselves into finance committees; the neighbours were appealed to for help, and in some cases as much as £20 was collected in a road. Many streets were wonderfully decorated from end to end with paper; teas were given to the children and sports organised in the roadway, while the fortunate possessors of pianos had dragged them to their front doors. The children have undoubtedly enjoyed one of the most novel treats of their lives.

### NEW HOSPITAL FOR GRAHAMSTOWN, SOUTH AFRICA.

The present Albany General Hospital, Grahamstown, is inadequate for the growing needs of the city and district. Our readers may remember that the proposed new hospital was originally estimated to cost £35,000, but the increase in the cost of materials and labour has raised the estimate to £62,000. The Administrator of the Cape Province has offered to provide £45,000 for the new building if the Albany Hospital Board will raise £10,000 privately towards the cost. It is fully expected that this amount could be raised. The Board itself has assets in the existing hospital and other property belonging to it. It is suggested that the hospital be called the Settlers Hospital as a memorial to the sturdy immigrants who left England in 1820 and peopled and civilised the Eastern Province of the Cape Colony. The centenary of their settling is to be commemorated next year in the Cape Province.

### MATERNITY WARDS IN WORKHOUSES.

Gradually there has been of late years a desire to remove maternity wards from the precincts of the workhouses. In a recent return to the Wandsworth Board of Guardians it was shown that in the Metropolitan area there are eleven maternity wards in workhouses and fourteen in infirmaries. The latter number will shortly be increased, for Bermondsey Board of Guardians has decided to remove the maternity ward from the workhouse in a densely populated district near Tooley Street to the more healthy infirmary at Rotherhithe. There will be little difficulty in making this desired alteration, which has the support of the newly appointed medical superintendent, Dr. R. C. Harkness. It will have the additional advantage of enabling the probationer nurses to take a course of training in midwifery, which was lacking at the infirmary.

### INCOME, GRANTS, AND ANNUAL SUBSCRIPTIONS.

That annual subscriptions can be raised by a definite appeal for them is shown by the fact that the Essex County Hospital is now receiving twice the amount which they yielded before the special appeal made in the spring. The increase is from £542 to £939. Whether this sum is worthy of Colchester and Essex is disputed. There are, of course arguments on both sides, but the voluntary system depends upon the support being equal to the need, and belief in that must inspire the effort which is necessary. The military patients have left and the War Office contributions have ceased. Now that more beds are available for civilians, it is for the hospital's supporters to maintain them. It is curious, by the way, to note that opinions differ as to the value of the War Office contributions. Some hospitals have found them adequate. But Mr. Shrewsbury Smith, the chairman of Worcester Infirmary, lately declared that, whereas the grant was 4s. 6d. for each soldier patient, the cost to the hospital was 4s. 11½d. It is true, of course, that a patient with a grant is more economical than one treated for nothing. But is this sufficient to explain the different effects which the War Office grants appear to have made on the various balance sheets?

### TWO LIVERPOOL HOSPITALS MERGE.

The amalagamation of Liverpool Infirmary for Children and the Royal Liverpool Country Hospital, already foreshadowed in these columns is now an accomplished fact. It is in the terms of the agreement that neither party shall be "considered to have absorbed the other." After the amalgamation had been unanimously adopted by separate and joint meetings of the governors, it was decided to extend each hospital and to appeal for the necessary funds. To complete the original scheme at Heswall £40,000 is required, while £20,000 is necessary to extend the infirmary's premises in Myrtle Street. One of the advantages of the amalgamation is to keep cases, many of whom require lengthy treatment, under the same doctors from their admission to their discharge. There will be two house committees, two matrons, a combined medical staff, and a single court of governors. To save the cost of replacing trustees, the two trusts are to be merged in an incorporated company. A new name for the joint institution will be chosen, in which it is hoped to retain the prefix Royal.

### PAY-WARDS FOR MATERNITY CASES.

The Samaritan Hospital for Women in Liverpool, which badly needs extension, is in the tantalising position of having acquired the necessary land while having to raise most of the £50,000 for the building. An interesting part of the plan is to provide accommodation for women of small means who cannot pay the expensive fees of the nursing home. Few of these take maternity cases, and those which do generally compare badly with the accommodation that a hospital can offer. We applaud this decision; it should also do much to popularise Mr. Charles Cain's appeal.

### PAY-WARDS VERSUS HOSPITAL TICKETS.

There is no voluntary hospital which still retains the ticket system of admission that does not suffer from twinges of conscience from time to time. Of the odious abuses attaching to the system we have often spoken, but there is good reason for a frequent return to the subject because the worst abuses are not the frightful things from which we all recoil but the evils which we have come to take for granted. Mr. J. P. Brown, the Mayor of Plymouth, has lately exclaimed against them in connection with the Devon and Cornwall Ear and Throat Hospital. One of the governors, Mr. John Adams, believes that the paying department which has been formed at the hospital will help to discountenance the tickets, because sensible people will prefer to pay something to avoid the trouble, delays, and disappointment entailed by a hunt after tickets. If this is so, it is only another argument in favour of pay-wards in the voluntary hospitals. The petty patronage conferred by tickets is not only cruel to waiting patients, but costly to the hospitals, since the cost of treatment is greater than the subscription which purchases a ticket.

### THE NEW PHASE AT EXETER.

The extension of the Royal Devon and Exeter Hospital has been decided on with the acceptance of the tender of £29,201 from Mr. E. C. Lea, of Okehampton. The plans, which have been drawn by Mr. E. H. Harbottle, include an extension of the children's ward, and such accommodation for discharged soldiers as may eventually be suitable for a nose, throat, and ear department. The Government promised a grant which Sir Channing Wills has doubled, and the £10,000 raised in this way may yet be increased by substantial help from the Red Cross. The cost of maintenance will be lightened by the payments made for the treatment of ex-Service men. A hopeful sign is the increase of subscriptions from workpeople, who have now begun to organise systematic contributions. Though the hospital possesses a collection sub-committee and a regular collector, the plan of sectional subscriptions from each class and area served by the hospital has yet to be carried out. In the past a collector went hither and thither to start or encourage local effort. The modern plan is for shops and factories, county and residential districts, to have their several funds on the lines of workpeople's contributions. Cambridge has done this successfully, and in the Midlands employers no less than their workers have organised separate funds. New buildings and a new financial policy must proceed side by side.

### A VETERAN SECRETARY RETIRES

Mr. J. Jones, the secretary of Croydon Hospital, has retired after forty-five years' service. Not only has the hospital grown from a small beginning to its present repute under Mr. Jones's hands but there is now no one associated with it who was at the hospital when Mr. Jones came. Indeed, he is but the second secretary, having succeeded Mr. W. Harland whose death was sudden. Croydon

Hospital has already trained several men who have made their mark later, and among the administrators we may note Mr. Harold Wigg, the present secretary of Hampstead General Hospital. In Mr. Jones's time the entire hospital has been rebuilt, the annual subscriptions have quadrupled, and the work done multiplied in every direction. Mr. Jones will remain at his post till September 30, and those who know Croydon Hospital can hardly fancy the place without his familiar presence.

### A JOINT APPEAL IN NORTH STAFFORDSHIRE.

The North Staffordshire Infirmary is a progressive institution which has done much to organise sectional subscriptions from employers and employed, and is to launch after the holidays the biggest appeal in its history. It is to be a joint appeal on behalf of the infirmary and the Cripples' Aid Society Hospital at Hartshill. It has been decided that the latter shall receive one-third of the total sum subscribed. Both hospitals are full and have long waiting lists. But the joint appeal is strengthened by the fact that the Crippled Children's Hospital in some sort completes the work of the infirmary, and the joining of the two institutions in a common appeal may lead in the future to a combination of the two institutions.

### THE GIPSIES IN THE WOOD.

At a recent meeting of the Southampton County Council reference was made to the difficulty of arranging for confinements among the gipsies who lived in the New Forest. The Health Committee now report that the Inspector of Midwives has visited East Boldre, and has gone into the question. It appears that the need is not so urgent as formerly, as the Aerodrome has been closed, and in consequence gipsies are not attracted to the place to the same extent. In any case, it is doubtful whether the women would be likely to use a cottage even were one available. If a caravan were provided it might solve the difficulty, as the gipsies would probably not raise any objections, and it could be moved from place to place.

### THE LATE CHAIRMAN OF THE SATURDAY FUND.

It is only eighteen months since the late Mr. Joseph Summers was first elected chairman of the Hospital Saturday Fund. He was re-elected last February, and the illness which overtook him in the middle of March was aggravated by the snowstorm of March 28, during which he was on duty superintending the cleaning of the streets in Southwark. He was an energetic borough engineer in Cambridge and elsewhere, and when in 1899 he came to London he began his connection with hospital work, first in Camberwell, and secondly on the Standing Committees of the Saturday Fund. He was sixty-four years old.

### ENDOWMENT OF MOTHERHOOD.

The Family Endowment Commission has laid before the National Birth-rate Commission in London that the State provide a regular weekly income for families with children under fifteen years of age. It is intended that the effect of this endowment will be to induce earlier marriages and will tend to remove the economic restriction of birth-rate.

### MASONIC HOSPITAL SERVICE.

St. John's Church, Leytonstone, was the scene of a somewhat remarkable gathering on July 6, the day appointed for National Thanksgiving for Peace, when members of the local Masonic Order attended a special service which had been arranged by the Vicar, Canon W. T. Brown. The sermon was preached by the Bishop of Chelmsford. A satisfactory result of the service was a collection of some £76 in aid of the Leyton War Memorial Hospital.

### BATTERSEA'S PEACE OFFERING.

An appeal is being made by the Governors of the Anti-Vivisection Hospital, Battersea, for £1,000, to be raised as a peace offering. This amount is required to equip the new out-patient department, and, unfortunately, until this sum is raised the patients will have to be content with roughing it in the present building. Upwards of 2,000 patients a month are treated in this department alone, with an average of 150 accident cases in addition. The out-patient department is sadly needed in Battersea, for in that thickly-populated borough the poor are predominant. In the interest of the patients it is essential that a thoroughly up-to-date out-patient department should be equipped and opened without delay.

### THE USE OF AUXILIARY HOSPITALS.

It is hoped to relieve the shortage of beds at the Royal Salop Infirmary by placing twenty or thirty children in the auxiliary hospital. We hope to see the plan more generally adopted, since many of the country houses used as auxiliary hospitals during the war could serve as convalescent homes, and therefore enable patients to be quickly passed through their mother institutions.

### THIS WEEK'S DRUG MARKET.

The market has barely recovered from the effect of the recent holiday, and, tempted by the hot weather, many dealers have prolonged their vacation. The result is that the market still has something of a holiday appearance, and for the most part buying is restricted to immediate requirements. Following on an advance in the price of crude iodine, quotations for resublimed iodine, iodoform, potassium iodide, and sodium iodide have been raised. The rise in the value of crude iodine had not been anticipated. On the other hand, the expected reduction in the price of morphine has now been made, but it is not so considerable as had been expected; codeine has also been reduced in price. Quotations for strychnine have been reduced, and English makers of caffeine have lowered their quotations. Atropine is also lower. The value of bromides is firmly maintained, and salicylic acid and sodium salicylate still have an upward price tendency. Menthol, Japanese peppermint oil, and Japanese refined camphor have quietened down.

